# Adaptation and Pathogenic Bacilli

**Published:** 1905-07-15

**Authors:** 


### Adaptation and Pathogenic Bacilli.

In an extremely graphic and lucid address recently delivered to the Canadian Association for the Prevention of Tuberculosis, Professor Adami presented an aspect of the interaction between pathogenic micro-organisms and the tissues of the body which is well worthy of careful attention. Everyone recognises and allows that the constituent cells of the body possess the capacity, within limits, to adjust themselves to new conditions. It is as a result of this capacity that the irruption of a host of invading bacteria is checked and the intruders destroyed. Further, it cannot be doubted that as generation has succeeded generation the ability of the tissue cells successfully to deal with the micro-organisms and toxins that cause infective diseases has been steadily increased. The organism has learned to adapt itself with a more accurate adjustment to the conditions established by the existence of these hurtful agents. In all this there is obvious room for congratulation and for confidence. But there is an aspect of the matter which is less satisfactory and which is too easily forgotten. The tissues, it is true, learn to offer greater resistance to individual species of micro-organisms, but these latter, in their turn, may possibly learn to attack with greater virulence. They, equally with the tissue cells, are living units and are therefore capable of adaptation or adjustment to environment. Such a conclusion may claim solid facts in its support. Thus, if a feebly pathogenic bacillus be introduced into the body of an animal, and when the disease so produced is in progress some of the tissue fluids be injected into a second animal of the same species, and this process be repeated through a series of animals, it will be found that the bacilli have become extremely virulent. At the commencement the disease had a mild form and ended in a cure, whilst at the end of the series the injected fluids promptly caused death. The bacilli, it is manifest, have adapted themselves, and indeed have more than adapted themselves, to the resistance of the organism. Viewed in this light the struggle of man with the infective agents responsible for a number of his diseases is seen to be a more complex process than at first sight appears. These agents are not fixed and unalterable in their properties. On the contrary, they possess the ability to meet an increased resistance with a more determined virulence, and as the one has become more resistant the other has become more toxic. That the balance of advantage has remained with the human body is probably due to the factors of union and co-operation which are possible, and indeed only possible, in a multicellular organism. Individual tissue-cells may be, and probably are, less capable of adaptation to new conditions than individual bacilli; but the sum of the reactions of the body when attacked may well be greater than the adaptive changes possible in the invading bacillus.

Another point to be considered is whether the increased virulence gained by a certain form of bacillus in conflict with one species of animal applies also to a different species. For example, have tubercle bacilli which have successfully adapted themselves to the conditions existing in oxen, an equal virulence in the tissues of the human body? The balance of evidence suggests a negative answer. Thus of two calves inoculated with equal doses of cultures of tubercle bacilli obtained from the cow and man respectively, the one receiving the bovine bacillus has a more serious and rapid form of the disease than the one infected from a human source. Indeed, a moderate dose of human bacilli is, as a matter of fact, successfully used as a vaccine to protect cattle from infection by their fellows with bovine tubercle. Such facts manifestly have a close bearing on the discussion whether or not tuberculosis is communicable from cattle to human beings. So far as they go they tend to indicate that this, whilst not impossible, cannot commonly or even frequently be the case. That tubercle bacilli having successfully adapted themselves to the conditions existing in bovine tissues must be equally virulent towards human tissues, is an argument which finds no support in our general knowledge of the laws wh[illegible] the condition the capacity for adaptation in micro-organisms.

